# Copula based prediction models: an application to an aortic regurgitation study

**DOI:** 10.1186/1471-2288-7-21

**Published:** 2007-06-16

**Authors:** Pranesh Kumar, Mohamed M Shoukri

**Affiliations:** 1 Department of Biostatistics, Epidemiology and Scientific Computing, King Faisal Specialist Hospital and Research Center, Riyadh 11211, Saudi Arabia; 2 Department of Mathematics, University of Northern British Columbia, Prince George, BC, Canada; 3 Department of Epidemiology and Biostatistics, Schulich School of Medicine, University of Western Ontario, London, Ontario, Canada

## Abstract

**Background:**

An important issue in prediction modeling of multivariate data is the measure of dependence structure. The use of Pearson's correlation as a dependence measure has several pitfalls and hence application of regression prediction models based on this correlation may not be an appropriate methodology. As an alternative, a copula based methodology for prediction modeling and an algorithm to simulate data are proposed.

**Methods:**

The method consists of introducing copulas as an alternative to the correlation coefficient commonly used as a measure of dependence. An algorithm based on the marginal distributions of random variables is applied to construct the *Archimedean *copulas. Monte Carlo simulations are carried out to replicate datasets, estimate prediction model parameters and validate them using Lin's concordance measure.

**Results:**

We have carried out a correlation-based regression analysis on data from 20 patients aged 17–82 years on pre-operative and post-operative ejection fractions after surgery and estimated the prediction model: Post-operative ejection fraction = - 0.0658 + 0.8403 (Pre-operative ejection fraction); p = 0.0008; 95% confidence interval of the slope coefficient (0.3998, 1.2808). From the exploratory data analysis, it is noted that both the pre-operative and post-operative ejection fractions measurements have slight departures from symmetry and are skewed to the left. It is also noted that the measurements tend to be widely spread and have shorter tails compared to normal distribution. Therefore predictions made from the correlation-based model corresponding to the pre-operative ejection fraction measurements in the lower range may not be accurate. Further it is found that the best approximated marginal distributions of pre-operative and post-operative ejection fractions (using q-q plots) are gamma distributions. The copula based prediction model is estimated as: Post -operative ejection fraction = - 0.0933 + 0.8907 × (Pre-operative ejection fraction); p = 0.00008 ; 95% confidence interval for slope coefficient (0.4810, 1.3003). For both models differences in the predicted post-operative ejection fractions in the lower range of pre-operative ejection measurements are considerably different and prediction errors due to copula model are smaller. To validate the copula methodology we have re-sampled with replacement fifty independent bootstrap samples and have estimated concordance statistics 0.7722 (p = 0.0224) for the copula model and 0.7237 (p = 0.0604) for the correlation model. The predicted and observed measurements are concordant for both models. The estimates of accuracy components are 0.9233 and 0.8654 for copula and correlation models respectively.

**Conclusion:**

Copula-based prediction modeling is demonstrated to be an appropriate alternative to the conventional correlation-based prediction modeling since the correlation-based prediction models are not appropriate to model the dependence in populations with asymmetrical tails. Proposed copula-based prediction model has been validated using the independent bootstrap samples.

## Background

Researchers, clinicians, and scientists are increasingly interested in the statistical models that have been designed to predict the occurrence of endpoint events given the diagnostic risk factors. The number and sophistication of cancer risk prediction models have grown rapidly over recent years and some researchers have expressed concerns as to whether they are always appropriately applied, correctly developed and rigorously evaluated. In 2004 the National Institutes of Health sponsored a workshop on *Cancer Risk Prediction Models: a Workshop on Development, Evaluation, and Application *in Washington D.C., USA. Experts associated with developing, evaluating, or using risk prediction models met to identify the strengths and limitations of cancer and genetic susceptibility prediction models currently in use and under development, in order to explore the methodological issues related to their development, evaluation and validation and also to identify the research priorities and resources needed to advance the field [1].

In this paper, a basic methodological issue of including the dependence parameter in the prediction model is considered. Pearson's linear correlation coefficient known as correlation is widely applied as a linear dependence measure. However, the correlation has several drawbacks and has a major impact on the accuracy of prediction models [2]. Correlation does not provide a complete description of the dependence structure even when there is a straight-line relationship between two random variables. Rather correlation is the canonical measure of the stochastic dependence used with normal (elliptical) distributions and is strongly affected by extreme endpoints. Independence of two random variables implies that they are uncorrelated but zero correlation, in general, does not imply independence unless distributions are multivariate normal. Furthermore, correlation is not invariant under non-linear strictly increasing transformations of random variables. Nonparametric measures of association like Kendall's rank correlation, Spearman's rank correlation and c-statistic are alternate measures of dependence which are more robust [3]. The kappa statistic is often used to measure the level of agreement when two categorical measurements of the same subjects are available. For an excellent review of dependence measures and their desirable properties, we refer to [2-4].

An alternative dependence measure is a copula which overcomes the limitations of correlation as a measure of dependence [5-8]. Use of copulas is a relatively new concept and has been applied in survival data analysis and actuaries [9,10]. Copulas are functions that join or couple multivariate distribution functions to their one-dimensional marginal distribution functions. Advantages of using copulas in modeling are (i) allowance to model both linear and non-linear dependence, (ii) arbitrary choice of a marginal distribution and (iii) capable of modeling extreme endpoints.

This paper describes the copula-based prediction modeling which can be employed as an alternative to the conventional correlation-based modeling in any multivariate clinical applications including risk-prediction. Implementation of copula based prediction approach is illustrated by analyzing data from patients with aortic regurgitation and corrective surgery [11].

## Methods

### Study example

The study example is adapted from an investigation [11] which enrolled 20 patients for isolated aortic regurgitation both before and after surgery and 20 patients for isolated mitral regurgitation. To correct the malfunctioning of the aortic valve, open heart surgery was performed and an artificial valve was sewn into the heart. Data collected were on patient's age, gender, NYHA class (amount of impairment in daily activities), heart rate (beats/minute), systolic blood pressure (mmHG), ejection fraction (fraction of blood in the left ventricle pumped out during a beat), EDVI-volume (ml/m^2^) of the left ventricle after the heart relaxes adjusted for body surface area (BSA), SVI-volume (ml/m^2^) of the left ventricle after the blood is pumped out adjusted for BSA, ESVI- volume (ml/m^2^) of the left ventricle pumped out during one cycle adjusted for BSA; ESVI=EDVI-SVI. These measurements were taken before and after valve replacement surgery. The patients were selected to have left ventricular volume overload, i. e., expanded EDVI. For the purpose of illustration, we have used data on post-operative ejection fraction and pre-operative ejection fraction from 20 patients with aortic regurgitation.

### What are copulas?

We denote the cumulative probability distribution of pre-operative ejection fraction (*X*) and post-operative ejection fraction (*Y*) by *H*(*x*, *y*) and marginal distributions of *X *and *Y *by *F*(*x*) and *G*(*y*) respectively. For uniform random variables *U *and *V *defined on [0,1] (by applying probability transforms *U *= *F*(*X*) and *V *= *G*(*Y*) to *X *and *Y*), there exists a bivariate copula function *C*(*u*, *v*) such that:

*H*(*x*, *y*) = Pr[*X *≤ *x*, *Y *≤ *y*] = *C *[*F*(*x*, *G*(*y*)] = *C*(*u*, *v*).

It is shown [2-4] that correlation *r *is only a limited description of the dependence between random variables except for the multivariate normal distribution where the correlation fully describes the dependence structure. If *F*(*x*) and *G*(*y*) are continuous then *C*(*u*, *v*) is unique otherwise *C*(*u*, *v*) is uniquely determined on range of *F*(*x*) × range of *G*(*y*). Since copulas link univariate marginals to their full multivariate distribution, an important feature of copulas is that any choice of marginal distributions can be used. Copulas are constructed on the assumption that marginal distributions are known or estimated from the data.

The two standard non-parametric dependence measures expressed in copula form are:

Kendall's Tau:τ=4∬I2C(u,v)dC(u,v)−1
 MathType@MTEF@5@5@+=feaafiart1ev1aaatCvAUfKttLearuWrP9MDH5MBPbIqV92AaeXatLxBI9gBaebbnrfifHhDYfgasaacH8akY=wiFfYdH8Gipec8Eeeu0xXdbba9frFj0=OqFfea0dXdd9vqai=hGuQ8kuc9pgc9s8qqaq=dirpe0xb9q8qiLsFr0=vr0=vr0dc8meaabaqaciaacaGaaeqabaqabeGadaaakeaafaqabeqacaaabaGaee4saSKaeeyzauMaeeOBa4MaeeizaqMaeeyyaeMaeeiBaWMaeeiBaWMaee4jaCIaee4CamNaeeiiaaIaeeivaqLaeeyyaeMaeeyDauNaeiOoaOdabaacciGae8hXdqNaeyypa0JaeGinaqZaa8GuaeaacqWGdbWqcqGGOaakcqWG1bqDcqGGSaalcqWG2bGDcqGGPaqkcqqGKbazcqWGdbWqcqGGOaakcqWG1bqDcqGGSaalcqWG2bGDcqGGPaqkcqGHsislcqaIXaqmaSqaaiabdMeajnaaCaaameqabaGaeGOmaidaaaWcbeqdcqGHRiI8cqGHRiI8aaaaaaa@5830@

Spearman's Rho:ρ=12∬I2C(u,v)dudv−3
 MathType@MTEF@5@5@+=feaafiart1ev1aaatCvAUfKttLearuWrP9MDH5MBPbIqV92AaeXatLxBI9gBaebbnrfifHhDYfgasaacH8akY=wiFfYdH8Gipec8Eeeu0xXdbba9frFj0=OqFfea0dXdd9vqai=hGuQ8kuc9pgc9s8qqaq=dirpe0xb9q8qiLsFr0=vr0=vr0dc8meaabaqaciaacaGaaeqabaqabeGadaaakeaafaqabeqacaaabaGaee4uamLaeeiCaaNaeeyzauMaeeyyaeMaeeOCaiNaeeyBa0MaeeyyaeMaeeOBa4Maee4jaCIaee4CamNaeeiiaaIaeeOuaiLaeeiAaGMaee4Ba8MaeiOoaOdabaacciGae8xWdiNaeyypa0JaeGymaeJaeGOmaiZaa8GuaeaacqWGdbWqcqGGOaakcqWG1bqDcqGGSaalcqWG2bGDcqGGPaqkcqqGKbazcqWG1bqDcqqGKbazcqWG2bGDcqGHsislcqaIZaWmaSqaaiabdMeajnaaCaaameqabaGaeGOmaidaaaWcbeqdcqGHRiI8cqGHRiI8aaaaaaa@5846@

The dependence measures *τ *and *ρ *calculated from the application data are used to estimate the copula parameter. It may be noted that the Pearson's correlation *r *cannot be expressed in copula form.

A special class of copulas known as *Archimedean *copulas [12] is defined by *C*(*u*, *v*) = *φ*^-1 ^[*φ *(*u*) + *φ *(*v*)] for all *u*, *v *∈ [0,1], where *φ *(*t*) is a generator function such that for all *t *∈ (0,1) *φ *(1) = 0 *φ *'(*t*) < 0, i.e., *φ *(*t*) is a decreasing function of *t *and *φ *"(*t*) ≥ 0, i.e., *φ *(*t*) is convex. One-parameter families of the *Archimedean *copulas with their generator functions are tabulated by Nelson [[6], p. 94].

From the copulas perspective multi-normal distribution has normal marginals and Gaussian (normal) copula dependence. Non-Gaussian copulas such as *t *and *Archimedean *can be used as an underlying dependence structure with any other non-normal marginals. Thus copulas provide flexibility in modeling datasets. Some examples of bivariate *Archimedean *copulas are given in Table 1.

**Table 1:** Bivariate *Archimedean *copulas, generator functions and Kendall's *τ*.

Copula	Generator *φ *(*t*)	*C*(*u*, *v*)	Kendall *τ*
Product (Independent)	-ln *t*	*u·v*	0
Clayton	(*t*^-*θ *^-1/*θ*, *θ *∈ [-1,∞)\{0}	(*u*^-*θ *^+ *v*^-*θ *^-1)^-1/*θ*^	*θ */(*θ *+ 2)
Gumbel	(-ln*t*)^*θ*^, *θ *∈ [1, ∞)	Exp [-{(-ln *u*)^*θ *^+ (-ln *v*)^*θ *^}^1/*θ *^]	(*θ *-1)/*θ*
Frank	−ln⁡e−tθ−1e−θ−1,θ∈R MathType@MTEF@5@5@+=feaafiart1ev1aaatCvAUfKttLearuWrP9MDH5MBPbIqV92AaeXatLxBI9gBaebbnrfifHhDYfgasaacH8akY=wiFfYdH8Gipec8Eeeu0xXdbba9frFj0=OqFfea0dXdd9vqai=hGuQ8kuc9pgc9s8qqaq=dirpe0xb9q8qiLsFr0=vr0=vr0dc8meaabaqaciaacaGaaeqabaqabeGadaaakeaacqGHsislcyGGSbaBcqGGUbGBdaWcaaqaaiabdwgaLnaaCaaaleqabaGaeyOeI0IaemiDaqhcciGae8hUdehaaOGaeyOeI0IaeGymaedabaGaemyzau2aaWbaaSqabeaacqGHsislcqWF4oqCaaGccqGHsislcqaIXaqmaaGaeiilaWIae8hUdeNaeyicI4SaemOuaifaaa@4338@	−1θln⁡[1+(e−uθ−1)(e−vθ−1)e−θ−1] MathType@MTEF@5@5@+=feaafiart1ev1aaatCvAUfKttLearuWrP9MDH5MBPbIqV92AaeXatLxBI9gBaebbnrfifHhDYfgasaacH8akY=wiFfYdH8Gipec8Eeeu0xXdbba9frFj0=OqFfea0dXdd9vqai=hGuQ8kuc9pgc9s8qqaq=dirpe0xb9q8qiLsFr0=vr0=vr0dc8meaabaqaciaacaGaaeqabaqabeGadaaakeaalmaalaaakeaajugqbiabgkHiTiabigdaXaGcbaacciqcLbuacqWF4oqCaaGagiiBaWMaeiOBa42cdaWadaGcbaqcLbuacqaIXaqmcqGHRaWklmaalaaakeaajugqbiabcIcaOiabdwgaLTWaaWbaaeqabaqcLbuacqGHsislcqWG1bqDcqWF4oqCaaGaeyOeI0IaeGymaeJaeiykaKIaeiikaGIaemyzau2cdaahaaqabeaajugqbiabgkHiTiabdAha2jab=H7aXbaacqGHsislcqaIXaqmcqGGPaqkaOqaaKqzafGaemyzau2cdaahaaqabeaajugqbiabgkHiTiab=H7aXbaacqGHsislcqaIXaqmaaaakiaawUfacaGLDbaaaaa@5502@	1−4θ[1−D1(θ)]∗ MathType@MTEF@5@5@+=feaafiart1ev1aaatCvAUfKttLearuWrP9MDH5MBPbIqV92AaeXatLxBI9gBaebbnrfifHhDYfgasaacH8akY=wiFfYdH8Gipec8Eeeu0xXdbba9frFj0=OqFfea0dXdd9vqai=hGuQ8kuc9pgc9s8qqaq=dirpe0xb9q8qiLsFr0=vr0=vr0dc8meaabaqaciaacaGaaeqabaqabeGadaaakeaacqaIXaqmcqGHsisldaWcaaqaaiabisda0aqaaGGaciab=H7aXbaadaWadaqaaiabigdaXiabgkHiTiabdseaenaaBaaaleaacqaIXaqmaeqaaOGaeiikaGIae8hUdeNaeiykaKcacaGLBbGaayzxaaWaaWbaaSqabeaacqGHxiIkaaaaaa@3BD1@

* *D*_*k*_(*x*) is the Debye function for any positive integer *k*, given by Dk(x)=kxk∫0xtket−1dt
MathType@MTEF@5@5@+=feaafiart1ev1aaatCvAUfKttLearuWrP9MDH5MBPbIqV92AaeXatLxBI9gBaebbnrfifHhDYfgasaacH8akY=wiFfYdH8Gipec8Eeeu0xXdbba9frFj0=OqFfea0dXdd9vqai=hGuQ8kuc9pgc9s8qqaq=dirpe0xb9q8qiLsFr0=vr0=vr0dc8meaabaqaciaacaGaaeqabaqabeGadaaakeaajugibiabdseaeTWaaSbaaeaajugibiabdUgaRbWcbeaajugibiabcIcaOiabdIha4jabcMcaPiabg2da9SWaaSaaaOqaaKqzGeGaem4AaSgakeaajugibiabdIha4TWaaWbaaeqabaqcLbsacqWGRbWAaaaaaSWaa8qCaOqaaSWaaSaaaOqaaKqzGeGaemiDaq3cdaahaaqabeaajugibiabdUgaRbaaaOqaaKqzGeGaemyzau2cdaahaaqabeaajugibiabdsha0baacqGHsislcqaIXaqmaaaaleaajugibiabicdaWaWcbaqcLbsacqWG4baEaiabgUIiYdGaeeizaqMaemiDaqhaaa@4E68@.

Sample versions of measures of dependence can be expressed in terms of empirical copula and corresponding empirical copula frequency function [6].

*Definition. Given *(*x*_*i*_, *y*_*i*_), *i *= 1, ...*n*, *a sample of size n from a bivariate distribution, the empirical copula is *C(in,jn)
 MathType@MTEF@5@5@+=feaafiart1ev1aaatCvAUfKttLearuWrP9MDH5MBPbIqV92AaeXatLxBI9gBaebbnrfifHhDYfgasaacH8akY=wiFfYdH8Gipec8Eeeu0xXdbba9frFj0=OqFfea0dXdd9vqai=hGuQ8kuc9pgc9s8qqaq=dirpe0xb9q8qiLsFr0=vr0=vr0dc8meaabaqaciaacaGaaeqabaqabeGadaaakeaacqWGdbWqcqGGOaakdaWcaaqaaiabdMgaPbqaaiabd6gaUbaacqGGSaaldaWcaaqaaiabdQgaQbqaaiabd6gaUbaacqGGPaqkaaa@35EF@ = *[Number of pairs *(*x*, *y*) *in the sample such that x *≤ *x*_(*i*)_*and y ≤ y*_(*j*)_*]/n, where x*_(*i*) _*and y *_(*j*)_, 1 ≤ *i*, *j *≤ *n*, *denote order statistics from the sample. The empirical copula frequency function is given by *c(in,jn)=1n
 MathType@MTEF@5@5@+=feaafiart1ev1aaatCvAUfKttLearuWrP9MDH5MBPbIqV92AaeXatLxBI9gBaebbnrfifHhDYfgasaacH8akY=wiFfYdH8Gipec8Eeeu0xXdbba9frFj0=OqFfea0dXdd9vqai=hGuQ8kuc9pgc9s8qqaq=dirpe0xb9q8qiLsFr0=vr0=vr0dc8meaabaqaciaacaGaaeqabaqabeGadaaakeaacqWGJbWycqGGOaakdaWcaaqaaiabdMgaPbqaaiabd6gaUbaacqGGSaaldaWcaaqaaiabdQgaQbqaaiabd6gaUbaacqGGPaqkcqGH9aqpdaWcaaqaaiabigdaXaqaaiabd6gaUbaaaaa@399A@*, if *(*x*_(*i*)_, *y*_(*j*)_) *is an element of the sample; Otherwise zero*.

### Simulation of bivariate Archimedean copulas

The following algorithm generates random variables (*U*, *V*) whose joint distribution is an *Archimedean *copula *C*(*u*, *v*) with generator function *φ *(*t*).

1. Generate two independent uniform random variables *p *and *q *on the interval [0,1].

2. Set *t *= *K*_*C*_^-1^(*q*) where *K*_*c *_is a copula function *C*(*u*, *v*).

3. Set *u *= *φ*^-1 ^[*p*·*φ *(*t*)] and *v *= *φ*^-1 ^[(1-*p*)·*φ *(*t*)].

4. Let *x *= *F*^-1^(*u*) and *y *= *F*^-1^(*v*).

5. Repeat *n *times steps 1 through 4 to generate *n *pairs of data (*x*_*i*_, *y*_*i*_), *i *= 1,..., *n*.

For implementing the algorithm, we perform the following steps [13]:

A. Kendall's rank correlation *τ *by the formula:

τ=(n2)−1∑i<jSign[(xi−xj)(yi−yj)].
 MathType@MTEF@5@5@+=feaafiart1ev1aaatCvAUfKttLearuWrP9MDH5MBPbIqV92AaeXatLxBI9gBaebbnrfifHhDYfgasaacH8akY=wiFfYdH8Gipec8Eeeu0xXdbba9frFj0=OqFfea0dXdd9vqai=hGuQ8kuc9pgc9s8qqaq=dirpe0xb9q8qiLsFr0=vr0=vr0dc8meaabaqaciaacaGaaeqabaqabeGadaaakeaaiiGacqWFepaDcqGH9aqpdaqadaqaauaabeqaceaaaeaacqWGUbGBaeaacqaIYaGmaaaacaGLOaGaayzkaaWaaWbaaSqabeaacqGHsislcqaIXaqmaaGcdaaeqbqaaiabbofatjabbMgaPjabbEgaNjabb6gaUnaadmaabaGaeiikaGIaemiEaG3aaSbaaSqaaiabdMgaPbqabaGccqGHsislcqWG4baEdaWgaaWcbaGaemOAaOgabeaakiabcMcaPiabcIcaOiabdMha5naaBaaaleaacqWGPbqAaeqaaOGaeyOeI0IaemyEaK3aaSbaaSqaaiabdQgaQbqabaGccqGGPaqkaiaawUfacaGLDbaaaSqaaiabdMgaPjabgYda8iabdQgaQbqab0GaeyyeIuoakiabc6caUaaa@54EA@

B. Copula parameter *θ *from *τ *.

C. Generator function *φ *(*t*).

D. First derivative of the generator function, *φ *'(*t*).

E. Inverse of the generator function, *φ*^-1^(*t*).

F. Copula function C(u,v)=KC(t)=t−ϕ(t)ϕ′(t)
 MathType@MTEF@5@5@+=feaafiart1ev1aaatCvAUfKttLearuWrP9MDH5MBPbIqV92AaeXatLxBI9gBaebbnrfifHhDYfgasaacH8akY=wiFfYdH8Gipec8Eeeu0xXdbba9frFj0=OqFfea0dXdd9vqai=hGuQ8kuc9pgc9s8qqaq=dirpe0xb9q8qiLsFr0=vr0=vr0dc8meaabaqaciaacaGaaeqabaqabeGadaaakeaacqWGdbWqcqGGOaakcqWG1bqDcqGGSaalcqWG2bGDcqGGPaqkcqGH9aqpcqWGlbWsdaWgaaWcbaGaem4qameabeaakiabcIcaOiabdsha0jabcMcaPiabg2da9iabdsha0jabgkHiTmaalaaabaacciGae8x1dOMaeiikaGIaemiDaqNaeiykaKcabaGaf8x1dOMbauaacqGGOaakcqWG0baDcqGGPaqkaaaaaa@4722@.

G. Inverse of copula function *K*_*C*_^-1^(*t*) In case no close form exists, solution is obtained by numerical root finding through the equation [t−ϕ(t)ϕ′(t)]−q
 MathType@MTEF@5@5@+=feaafiart1ev1aaatCvAUfKttLearuWrP9MDH5MBPbIqV92AaeXatLxBI9gBaebbnrfifHhDYfgasaacH8akY=wiFfYdH8Gipec8Eeeu0xXdbba9frFj0=OqFfea0dXdd9vqai=hGuQ8kuc9pgc9s8qqaq=dirpe0xb9q8qiLsFr0=vr0=vr0dc8meaabaqaciaacaGaaeqabaqabeGadaaakeaadaWadaqaaiabdsha0jabgkHiTmaalaaabaacciGae8x1dOMaeiikaGIaemiDaqNaeiykaKcabaGaf8x1dOMbauaacqGGOaakcqWG0baDcqGGPaqkaaaacaGLBbGaayzxaaGaeyOeI0IaemyCaehaaa@3D50@.

H. *u *= *φ*^-1 ^[*p*·*φ *(*t*)] and *v *= *φ*^-1 ^[(1-*p*)·*φ *(*t*)].

For ready reference algorithm implementation steps for some commonly applied *Archimedean *copulas are worked out and are given in Table 2.

**Table 2:** Algorithm steps for the *Archimedean *copulas.

Step	Clayton	Gumbel	Frank
B: *θ*	2*τ */(1-*τ*)	1/(1-*τ*)	No closed form
C:*φ*(*t*)	(*t*^-*θ *^-1)/*θ*	(-ln *t*)^*θ*^	T:=−ln⁡e−tθ−1e−θ−1 MathType@MTEF@5@5@+=feaafiart1ev1aaatCvAUfKttLearuWrP9MDH5MBPbIqV92AaeXatLxBI9gBaebbnrfifHhDYfgasaacH8akY=wiFfYdH8Gipec8Eeeu0xXdbba9frFj0=OqFfea0dXdd9vqai=hGuQ8kuc9pgc9s8qqaq=dirpe0xb9q8qiLsFr0=vr0=vr0dc8meaabaqaciaacaGaaeqabaqabeGadaaakeaacqWGubavcqGG6aGocqGH9aqpcqGHsislcyGGSbaBcqGGUbGBdaWcaaqaaiabdwgaLnaaCaaaleqabaGaeyOeI0IaemiDaqhcciGae8hUdehaaOGaeyOeI0IaeGymaedabaGaemyzau2aaWbaaSqabeaacqGHsislcqWF4oqCaaGccqGHsislcqaIXaqmaaaaaa@4129@
D:*φ *'(*t*)	-*θ*·*t*^-*θ*-1^	-*θ*(ln *t*)^*θ *-1^/*t*	*θ */(1-*e*^*tθ*^)
E:*φ *^-1^(*t*)	(1 + t)^-1/*θ*^	exp((-*t*)^*t*/*θ*^)	−ln⁡(1−e−t+e−t−θ)θ MathType@MTEF@5@5@+=feaafiart1ev1aaatCvAUfKttLearuWrP9MDH5MBPbIqV92AaeXatLxBI9gBaebbnrfifHhDYfgasaacH8akY=wiFfYdH8Gipec8Eeeu0xXdbba9frFj0=OqFfea0dXdd9vqai=hGuQ8kuc9pgc9s8qqaq=dirpe0xb9q8qiLsFr0=vr0=vr0dc8meaabaqaciaacaGaaeqabaqabeGadaaakeaacqGHsisldaWcaaqaaiGbcYgaSjabc6gaUnaabmaabaGaeGymaeJaeyOeI0Iaemyzau2aaWbaaSqabeaacqGHsislcqWG0baDaaGccqGHRaWkcqWGLbqzdaahaaWcbeqaaiabgkHiTiabdsha0jabgkHiTGGaciab=H7aXbaaaOGaayjkaiaawMcaaaqaaiabeI7aXbaaaaa@40E7@
F:*K*_*c*_	*t *- ((*t*^1+*θ *^- *t*)/*θ*)	*t *-(*t*ln *t*/*θ*)	t−(etθ−1)θln⁡e−tθ−1e−θ−1 MathType@MTEF@5@5@+=feaafiart1ev1aaatCvAUfKttLearuWrP9MDH5MBPbIqV92AaeXatLxBI9gBaebbnrfifHhDYfgasaacH8akY=wiFfYdH8Gipec8Eeeu0xXdbba9frFj0=OqFfea0dXdd9vqai=hGuQ8kuc9pgc9s8qqaq=dirpe0xb9q8qiLsFr0=vr0=vr0dc8meaabaqaciaacaGaaeqabaqabeGadaaakeaajugqbiabdsha0jabgkHiTSWaaSaaaOqaaSWaaeWaaOqaaKqzafGaemyzau2cdaahaaqabeaajugqbiabdsha0HGaciab=H7aXbaacqGHsislcqaIXaqmaOGaayjkaiaawMcaaaqaaKqzafGae8hUdehaaiGbcYgaSjabc6gaUTWaaSaaaOqaaKqzafGaemyzau2cdaahaaqabeaajugqbiabgkHiTiabdsha0jab=H7aXbaacqGHsislcqaIXaqmaOqaaKqzafGaemyzau2cdaahaaqabeaajugqbiabgkHiTiab=H7aXbaacqGHsislcqaIXaqmaaaaaa@4EE7@
G:*K*^-1^*c*	No closed form	No closed form	No closed form
H:(uv) MathType@MTEF@5@5@+=feaafiart1ev1aaatCvAUfKttLearuWrP9MDH5MBPbIqV92AaeXatLxBI9gBaebbnrfifHhDYfgasaacH8akY=wiFfYdH8Gipec8Eeeu0xXdbba9frFj0=OqFfea0dXdd9vqai=hGuQ8kuc9pgc9s8qqaq=dirpe0xb9q8qiLsFr0=vr0=vr0dc8meaabaqaciaacaGaaeqabaqabeGadaaakeaacqqGibascqGG6aGodaqadaqaauaabeqaceaaaeaacqWG1bqDaeaacqWG2bGDaaaacaGLOaGaayzkaaaaaa@333D@	((1+p⋅(t−θ−1))−1/θ(1+(1−p)⋅(t−θ−1))−1/θ) MathType@MTEF@5@5@+=feaafiart1ev1aaatCvAUfKttLearuWrP9MDH5MBPbIqV92AaeXatLxBI9gBaebbnrfifHhDYfgasaacH8akY=wiFfYdH8Gipec8Eeeu0xXdbba9frFj0=OqFfea0dXdd9vqai=hGuQ8kuc9pgc9s8qqaq=dirpe0xb9q8qiLsFr0=vr0=vr0dc8meaabaqaciaacaGaaeqabaqabeGadaaakeaadaqadaqaauaabeqaceaaaeaadaqadaqaaiabigdaXiabgUcaRiabdchaWjabgwSixlabcIcaOiabdsha0naaCaaaleqabaGaeyOeI0ccciGae8hUdehaaOGaeyOeI0IaeGymaeJaeiykaKcacaGLOaGaayzkaaWaaWbaaSqabeaacqGHsislcqaIXaqmcqGGVaWlcqWF4oqCaaaakeaadaqadaqaaiabigdaXiabgUcaRiabcIcaOiabigdaXiabgkHiTiabdchaWjabcMcaPiabgwSixlabcIcaOiabdsha0naaCaaaleqabaGaeyOeI0Iae8hUdehaaOGaeyOeI0IaeGymaeJaeiykaKcacaGLOaGaayzkaaWaaWbaaSqabeaacqGHsislcqaIXaqmcqGGVaWlcqWF4oqCaaaaaaGccaGLOaGaayzkaaaaaa@58F9@	(exp⁡(−(p⋅(−ln⁡t)θ)1/θ)exp⁡(−{(1−p)⋅(−ln⁡t)θ}1/θ)) MathType@MTEF@5@5@+=feaafiart1ev1aaatCvAUfKttLearuWrP9MDH5MBPbIqV92AaeXatLxBI9gBaebbnrfifHhDYfgasaacH8akY=wiFfYdH8Gipec8Eeeu0xXdbba9frFj0=OqFfea0dXdd9vqai=hGuQ8kuc9pgc9s8qqaq=dirpe0xb9q8qiLsFr0=vr0=vr0dc8meaabaqaciaacaGaaeqabaqabeGadaaakeaadaqadaqaauaabeqaceaaaeaacyGGLbqzcqGG4baEcqGGWbaCdaqadaqaaiabgkHiTiabcIcaOiabdchaWjabgwSixlabcIcaOiabgkHiTiGbcYgaSjabc6gaUjabdsha0jabcMcaPmaaCaaaleqabaacciGae8hUdehaaOGaeiykaKYaaWbaaSqabeaacqaIXaqmcqGGVaWlcqWF4oqCaaaakiaawIcacaGLPaaaaeaacyGGLbqzcqGG4baEcqGGWbaCdaqadaqaaiabgkHiTiabcUha7jabcIcaOiabigdaXiabgkHiTiabdchaWjabcMcaPiabgwSixlabcIcaOiabgkHiTiGbcYgaSjabc6gaUjabdsha0jabcMcaPmaaCaaaleqabaGae8hUdehaaOGaeiyFa03aaWbaaSqabeaacqaIXaqmcqGGVaWlcqWF4oqCaaaakiaawIcacaGLPaaaaaaacaGLOaGaayzkaaaaaa@6441@	(−ln⁡(1−e−pT+e−pT−θ)/θ−ln⁡(1−e−(1−p)T+e−(1−p)T−θ)/θ) MathType@MTEF@5@5@+=feaafiart1ev1aaatCvAUfKttLearuWrP9MDH5MBPbIqV92AaeXatLxBI9gBaebbnrfifHhDYfgasaacH8akY=wiFfYdH8Gipec8Eeeu0xXdbba9frFj0=OqFfea0dXdd9vqai=hGuQ8kuc9pgc9s8qqaq=dirpe0xb9q8qiLsFr0=vr0=vr0dc8meaabaqaciaacaGaaeqabaqabeGadaaakeaadaqadaqaauaabeqaceaaaeaacqGHsislcyGGSbaBcqGGUbGBdaqadaqaaiabigdaXiabgkHiTiabdwgaLnaaCaaaleqabaGaeyOeI0IaemiCaaNaemivaqfaaOGaey4kaSIaemyzau2aaWbaaSqabeaacqGHsislcqWGWbaCcqWGubavcqGHsisliiGacqWF4oqCaaaakiaawIcacaGLPaaacqGGVaWlcqWF4oqCaeaacqGHsislcyGGSbaBcqGGUbGBdaqadaqaaiabigdaXiabgkHiTiabdwgaLnaaCaaaleqabaGaeyOeI0IaeiikaGIaeGymaeJaeyOeI0IaemiCaaNaeiykaKIaemivaqfaaOGaey4kaSIaemyzau2aaWbaaSqabeaacqGHsislcqGGOaakcqaIXaqmcqGHsislcqWGWbaCcqGGPaqkcqWGubavcqGHsislcqWF4oqCaaaakiaawIcacaGLPaaacqGGVaWlcqWF4oqCaaaacaGLOaGaayzkaaaaaa@6410@

### Evaluating copulas

The first step in modeling and simulation is to identify the appropriate copula form. To identify the most appropriate copula for the given application data set (*x*_*i*_, *y*_*i*_), *i *= 1,...*n*, we follow the procedure [3,7]:

1. Calculate the non-parametric Kendall's rank correlation *τ *using the formula in equation (4).

2. Construct an empirical copula function *K*_*E*_(*t*) as follows:

i. Determine the pseudo observations *T*_*i *_= {Number of(*x*_*j *_<*x*_*i*_) such that *x*_*j *_≤ *x*_*i *_and *y*_*j *_≤ *y*_*i*_}/(*n*-1).

ii. The empirical copula *K*_*E*_(*t*) = proportion of *T*_*i*_'*s *≤ *t*, 0 ≤ *t *≤ 1.

In non-mathematical terms, it means that for all pairs of subjects in which the Y-value for a given subject is lower (or higher) than the Y-value of a second subject, for what proportion of X-values does the first subject also have a lower (or higher) value?

3. Construct the Archimedean copula function KC(t)=t−ϕ(t)ϕ′(t)
 MathType@MTEF@5@5@+=feaafiart1ev1aaatCvAUfKttLearuWrP9MDH5MBPbIqV92AaeXatLxBI9gBaebbnrfifHhDYfgasaacH8akY=wiFfYdH8Gipec8Eeeu0xXdbba9frFj0=OqFfea0dXdd9vqai=hGuQ8kuc9pgc9s8qqaq=dirpe0xb9q8qiLsFr0=vr0=vr0dc8meaabaqaciaacaGaaeqabaqabeGadaaakeaacqWGlbWsdaWgaaWcbaGaem4qameabeaakiabcIcaOiabdsha0jabcMcaPiabg2da9iabdsha0jabgkHiTmaalaaabaacciGae8x1dOMaeiikaGIaemiDaqNaeiykaKcabaGaf8x1dOMbauaacqGGOaakcqWG0baDcqGGPaqkaaaaaa@3F93@.

In order to select the Archimedean copula that best fits the application data, we use a probability – plot or choose that copula which minimizes the non-parametric distance measure *DM*: ∫ [*K*_*c*_(*t*) - *K*_*E*_(*t*)]^2^*dK*_*E*_(*t*). For simulating bivariate *Archimedean *copulas, we refer to [14].

Further it may be worth exploring the connections of copulas to other non-parametric association statistics like c-statistic which are defined in terms of the concordant (C) and discordant (D) pairs. One such relationship is easily seen to exist between the Gumbel copula parameter *θ *and the concordant and discordant pairs. The Kendall's rank correlation *τ *in terms of (C, D) pairs is *τ *= 2(*C*-*D*)/*n*(*n*-1) and the Gumbel copula parameter *θ *and Kendall's rank correlation are related by *τ *= (*θ *-1)/*θ *. Thus it is easily seen that θ=[1−2(C−D)n(n−1)]−1
 MathType@MTEF@5@5@+=feaafiart1ev1aaatCvAUfKttLearuWrP9MDH5MBPbIqV92AaeXatLxBI9gBaebbnrfifHhDYfgasaacH8akY=wiFfYdH8Gipec8Eeeu0xXdbba9frFj0=OqFfea0dXdd9vqai=hGuQ8kuc9pgc9s8qqaq=dirpe0xb9q8qiLsFr0=vr0=vr0dc8meaabaqaciaacaGaaeqabaqabeGadaaakeaaiiGacqWF4oqCcqGH9aqpdaWadaqaaiabigdaXiabgkHiTmaalaaabaGaeGOmaiJaeiikaGIaem4qamKaeyOeI0IaemiraqKaeiykaKcabaGaemOBa4MaeiikaGIaemOBa4MaeyOeI0IaeGymaeJaeiykaKcaaaGaay5waiaaw2faamaaCaaaleqabaGaeyOeI0IaeGymaedaaaaa@4162@.

### Validating the prediction model

To validate a prediction model, joint assessment of precision and accuracy is required. In [15], Lin proposed the concordance correlation coefficient ρyy^
 MathType@MTEF@5@5@+=feaafiart1ev1aaatCvAUfKttLearuWrP9MDH5MBPbIqV92AaeXatLxBI9gBaebbnrfifHhDYfgasaacH8akY=wiFfYdH8Gipec8Eeeu0xXdbba9frFj0=OqFfea0dXdd9vqai=hGuQ8kuc9pgc9s8qqaq=dirpe0xb9q8qiLsFr0=vr0=vr0dc8meaabaqaciaacaGaaeqabaqabeGadaaakeaaiiGacqWFbpGCdaWgaaWcbaGaemyEaKNafmyEaKNbaKaaaeqaaaaa@31A5@ to evaluate the agreement (reproducibility) between two sets of observed (y) and predicted (y) data. The concordance correlation ρyy^
 MathType@MTEF@5@5@+=feaafiart1ev1aaatCvAUfKttLearuWrP9MDH5MBPbIqV92AaeXatLxBI9gBaebbnrfifHhDYfgasaacH8akY=wiFfYdH8Gipec8Eeeu0xXdbba9frFj0=OqFfea0dXdd9vqai=hGuQ8kuc9pgc9s8qqaq=dirpe0xb9q8qiLsFr0=vr0=vr0dc8meaabaqaciaacaGaaeqabaqabeGadaaakeaaiiGacqWFbpGCdaWgaaWcbaGaemyEaKNafmyEaKNbaKaaaeqaaaaa@31A5@ is defined as:

ρyy^=2σyy^/[σy 2+σy^ 2+(μy−μy^)2],
 MathType@MTEF@5@5@+=feaafiart1ev1aaatCvAUfKttLearuWrP9MDH5MBPbIqV92AaeXatLxBI9gBamXvP5wqSXMqHnxAJn0BKvguHDwzZbqegyvzYrwyUfgarqqtubsr4rNCHbGeaGqiA8vkIkVAFgIELiFeLkFeLk=iY=Hhbbf9v8qqaqFr0xc9pk0xbba9q8WqFfeaY=biLkVcLq=JHqVepeea0=as0db9vqpepesP0xe9Fve9Fve9GapdbaqaaeGacaGaaiaabeqaamqadiabaaGcbaacciGae8xWdi3aaSbaaSqaaiabdMha5jqbdMha5zaajaaabeaakiabg2da9iabikdaYiab=n8aZnaaBaaaleaacqWG5bqEcuWG5bqEgaqcaaqabaGccqGGVaWlcqGGBbWwcqWFdpWCdaqhaaWcbaGaeeyEaKhabaGaeeiiaaIaeeOmaidaaOGaey4kaSIae83Wdm3aa0baaSqaaiqbbMha5zaajaaabaGaeeiiaaIaeeOmaidaaOGaey4kaSIaeiikaGIae8hVd02aaSbaaSqaaiabdMha5bqabaGccqGHsislcqWF8oqBdaWgaaWcbaGafmyEaKNbaKaaaeqaaOGaeiykaKYaaWbaaSqabeaacqaIYaGmaaGccqGGDbqxcqGGSaalaaa@63D6@

where *μ*_*y *_and μy^
 MathType@MTEF@5@5@+=feaafiart1ev1aaatCvAUfKttLearuWrP9MDH5MBPbIqV92AaeXatLxBI9gBaebbnrfifHhDYfgasaacH8akY=wiFfYdH8Gipec8Eeeu0xXdbba9frFj0=OqFfea0dXdd9vqai=hGuQ8kuc9pgc9s8qqaq=dirpe0xb9q8qiLsFr0=vr0=vr0dc8meaabaqaciaacaGaaeqabaqabeGadaaakeaaiiGacqWF8oqBdaWgaaWcbaGafmyEaKNbaKaaaeqaaaaa@3020@ are means of (*y*) and (y^
 MathType@MTEF@5@5@+=feaafiart1ev1aaatCvAUfKttLearuWrP9MDH5MBPbIqV92AaeXatLxBI9gBaebbnrfifHhDYfgasaacH8akY=wiFfYdH8Gipec8Eeeu0xXdbba9frFj0=OqFfea0dXdd9vqai=hGuQ8kuc9pgc9s8qqaq=dirpe0xb9q8qiLsFr0=vr0=vr0dc8meaabaqaciaacaGaaeqabaqabeGadaaakeaacuWG5bqEgaqcaaaa@2E37@), *σ*_*y*_^2 ^and σy^ 2
 MathType@MTEF@5@5@+=feaafiart1ev1aaatCvAUfKttLearuWrP9MDH5MBPbIqV92AaeXatLxBI9gBamXvP5wqSXMqHnxAJn0BKvguHDwzZbqegyvzYrwyUfgarqqtubsr4rNCHbGeaGqiA8vkIkVAFgIELiFeLkFeLk=iY=Hhbbf9v8qqaqFr0xc9pk0xbba9q8WqFfeaY=biLkVcLq=JHqVepeea0=as0db9vqpepesP0xe9Fve9Fve9GapdbaqaaeGacaGaaiaabeqaamqadiabaaGcbaacciGae83Wdm3aa0baaSqaaiqbbMha5zaajaaabaGaeeiiaaIaeeOmaidaaaaa@421D@ denote variances of (*y*) and (y^
 MathType@MTEF@5@5@+=feaafiart1ev1aaatCvAUfKttLearuWrP9MDH5MBPbIqV92AaeXatLxBI9gBaebbnrfifHhDYfgasaacH8akY=wiFfYdH8Gipec8Eeeu0xXdbba9frFj0=OqFfea0dXdd9vqai=hGuQ8kuc9pgc9s8qqaq=dirpe0xb9q8qiLsFr0=vr0=vr0dc8meaabaqaciaacaGaaeqabaqabeGadaaakeaacuWG5bqEgaqcaaaa@2E37@) and σyy^
 MathType@MTEF@5@5@+=feaafiart1ev1aaatCvAUfKttLearuWrP9MDH5MBPbIqV92AaeXatLxBI9gBaebbnrfifHhDYfgasaacH8akY=wiFfYdH8Gipec8Eeeu0xXdbba9frFj0=OqFfea0dXdd9vqai=hGuQ8kuc9pgc9s8qqaq=dirpe0xb9q8qiLsFr0=vr0=vr0dc8meaabaqaciaacaGaaeqabaqabeGadaaakeaaiiGacqWFdpWCdaWgaaWcbaGaemyEaKNafmyEaKNbaKaaaeqaaaaa@31A8@ is the population covariance between (*y*) and (y^
 MathType@MTEF@5@5@+=feaafiart1ev1aaatCvAUfKttLearuWrP9MDH5MBPbIqV92AaeXatLxBI9gBaebbnrfifHhDYfgasaacH8akY=wiFfYdH8Gipec8Eeeu0xXdbba9frFj0=OqFfea0dXdd9vqai=hGuQ8kuc9pgc9s8qqaq=dirpe0xb9q8qiLsFr0=vr0=vr0dc8meaabaqaciaacaGaaeqabaqabeGadaaakeaacuWG5bqEgaqcaaaa@2E37@). The concordance correlation ρyy^
 MathType@MTEF@5@5@+=feaafiart1ev1aaatCvAUfKttLearuWrP9MDH5MBPbIqV92AaeXatLxBI9gBaebbnrfifHhDYfgasaacH8akY=wiFfYdH8Gipec8Eeeu0xXdbba9frFj0=OqFfea0dXdd9vqai=hGuQ8kuc9pgc9s8qqaq=dirpe0xb9q8qiLsFr0=vr0=vr0dc8meaabaqaciaacaGaaeqabaqabeGadaaakeaaiiGacqWFbpGCdaWgaaWcbaGaemyEaKNafmyEaKNbaKaaaeqaaaaa@31A5@ is a product of precision ryy^
 MathType@MTEF@5@5@+=feaafiart1ev1aaatCvAUfKttLearuWrP9MDH5MBPbIqV92AaeXatLxBI9gBaebbnrfifHhDYfgasaacH8akY=wiFfYdH8Gipec8Eeeu0xXdbba9frFj0=OqFfea0dXdd9vqai=hGuQ8kuc9pgc9s8qqaq=dirpe0xb9q8qiLsFr0=vr0=vr0dc8meaabaqaciaacaGaaeqabaqabeGadaaakeaacqWGYbGCdaWgaaWcbaGaemyEaKNafmyEaKNbaKaaaeqaaaaa@314B@ (correlation between *Y *and Y^
 MathType@MTEF@5@5@+=feaafiart1ev1aaatCvAUfKttLearuWrP9MDH5MBPbIqV92AaeXatLxBI9gBaebbnrfifHhDYfgasaacH8akY=wiFfYdH8Gipec8Eeeu0xXdbba9frFj0=OqFfea0dXdd9vqai=hGuQ8kuc9pgc9s8qqaq=dirpe0xb9q8qiLsFr0=vr0=vr0dc8meaabaqaciaacaGaaeqabaqabeGadaaakeaacuWGzbqwgaqcaaaa@2DF7@) and accuracy γyy^
 MathType@MTEF@5@5@+=feaafiart1ev1aaatCvAUfKttLearuWrP9MDH5MBPbIqV92AaeXatLxBI9gBaebbnrfifHhDYfgasaacH8akY=wiFfYdH8Gipec8Eeeu0xXdbba9frFj0=OqFfea0dXdd9vqai=hGuQ8kuc9pgc9s8qqaq=dirpe0xb9q8qiLsFr0=vr0=vr0dc8meaabaqaciaacaGaaeqabaqabeGadaaakeaaiiGacqWFZoWzdaWgaaWcbaGaemyEaKNafmyEaKNbaKaaaeqaaaaa@318C@, where accuracy

γyy^=2σyσy^/[σy 2+σy^ 2+(μy−μy^)2].
 MathType@MTEF@5@5@+=feaafiart1ev1aaatCvAUfKttLearuWrP9MDH5MBPbIqV92AaeXatLxBI9gBamXvP5wqSXMqHnxAJn0BKvguHDwzZbqegyvzYrwyUfgarqqtubsr4rNCHbGeaGqiA8vkIkVAFgIELiFeLkFeLk=iY=Hhbbf9v8qqaqFr0xc9pk0xbba9q8WqFfeaY=biLkVcLq=JHqVepeea0=as0db9vqpepesP0xe9Fve9Fve9GapdbaqaaeGacaGaaiaabeqaamqadiabaaGcbaacciGae83SdC2aaSbaaSqaaiabdMha5jqbdMha5zaajaaabeaakiabg2da9iabikdaYiab=n8aZnaaBaaaleaacqWG5bqEaeqaaOGae83Wdm3aaSbaaSqaaiqbdMha5zaajaaabeaakiabc+caVmaadmaabaGae83Wdm3aa0baaSqaaiabbMha5bqaaiabbccaGiabbkdaYaaakiabgUcaRiab=n8aZnaaDaaaleaacuqG5bqEgaqcaaqaaiabbccaGiabbkdaYaaakiabgUcaRiabcIcaOiab=X7aTnaaBaaaleaacqWG5bqEaeqaaOGaeyOeI0Iae8hVd02aaSbaaSqaaiqbdMha5zaajaaabeaakiabcMcaPmaaCaaaleqabaGaeGOmaidaaaGccaGLBbGaayzxaaGaeiOla4caaa@6527@

The estimation of concordance correlation ρyy^
 MathType@MTEF@5@5@+=feaafiart1ev1aaatCvAUfKttLearuWrP9MDH5MBPbIqV92AaeXatLxBI9gBaebbnrfifHhDYfgasaacH8akY=wiFfYdH8Gipec8Eeeu0xXdbba9frFj0=OqFfea0dXdd9vqai=hGuQ8kuc9pgc9s8qqaq=dirpe0xb9q8qiLsFr0=vr0=vr0dc8meaabaqaciaacaGaaeqabaqabeGadaaakeaaiiGacqWFbpGCdaWgaaWcbaGaemyEaKNafmyEaKNbaKaaaeqaaaaa@31A5@ and its asymptotic sampling distribution are discussed in Lin [15].

## Results

### Application

**Figure 1:**
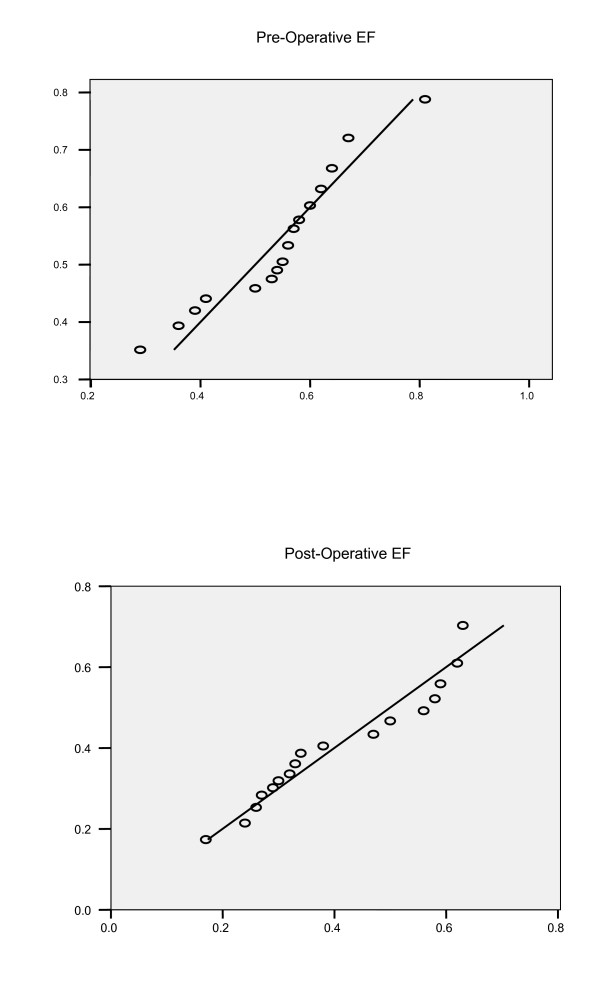
Quantile Plots of pre-operative and post-operative ejection fractions.

**Figure 2:**
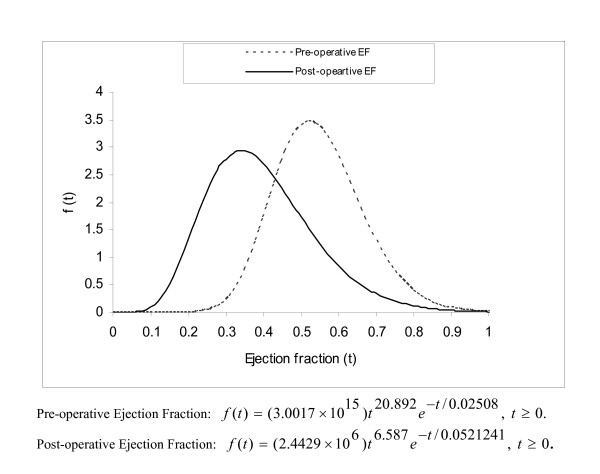
Marginal distributions of pre-operative and post-operative ejection fractions.

The measurements on pre-operative and post-operative ejection fraction from 20 patients with aortic regurgitation including their ages are given in Table 3. The exploratory data analysis indicates that both the pre-operative and post-operative ejection fractions: (i) have slight departures from symmetry, (ii) are skewed in left tails (skewness coefficients being -0.3340 and 0.3730, respectively), (iii) have tendency of measurements to cluster less and (iv) have shorter tails (kurtosis coefficients being 0.9680 and -1.2540, respectively). Since both the pre-operative and post-operative measurements show deviations from normal distributions, probability plots for normal, gamma and Weibull distributions were fitted. From the plots, gamma distributions are found to be the best fit since data points clustered mostly around a straight line for the gamma fit. Estimates of parameters of marginal distributions of pre-operative and post-operative measurements are given in Table 3. Probability plots are graphed in Figure 1 and estimated marginal distributions are given in Figure 2.

**Table 3:** Data from patients with aortic regurgitation.

Case	Age(years) Sex	NYHA Class	Pre-operative Ejection Fraction	Post-operative Ejection Fraction
1	33 M	I	0.54	0.38
2	36 M	I	0.64	0.58
3	37 M	I	0.50	0.27
4	38 M	I	0.41	0.17
5	38 M	I	0.53	0.47
6	54 M	I	0.56	0.50
7	56 F	I	0.81	0.56
8	70 M	I	0.67	0.59
9	22 M	II	0.57	0.33
10	28 F	II	0.58	0.32
11	40 M	II	0.62	0.47
12	48 F	II	0.36	0.24
13	42 F	III	0.64	0.63
14	57 M	III	0.60	0.33
15	61 M	III	0.56	0.34
16	64 M	III	0.60	0.30
17	61 M	IV	0.55	0.62
18	62 M	IV	0.56	0.29
19	64 M	IV	0.39	0.26
20	65 M	IV	0.29	0.26
Mean	49		0.5490	0.3955
Standard Deviation	14		0.1173	0.1436
Gamma Distribution	Shape		21.8920	7.5870
	Scale		39.8770	19.1850

There is an evidence of significant association between the pre-operative and post-operative ejection fractions since the Pearson's correlation coefficient *r *= 0.6870 (*p < 0.0010*), Kendall's rank correlation *τ *= 0.5050 (*p < 0.0020*) and Spearman's rank correlation *ρ *= 0.6970 (*p < 0.0010*).

For predicting the post-operative ejection fraction of a patient after surgery given pre-operative ejection fraction measurement, we have estimated the conventional prediction regression model using correlation coefficient:

Post-operative ejection fraction^correlation ^= -0.0658 + 0.8403 × (Pre-operative ejection fraction); p = 0.0008; 95% confidence interval: (0.3998, 1.2808).

The p-value indicates that the estimated model is useful in predicting the post-operative ejection fraction of a patient given the pre-operative ejection fraction. However, predictions made in the lower range of pre-operative ejection fractions may not be accurate because of the skewness exhibited by data in the left tail. As an alternative a copula-based prediction model is discussed below.

### Simulation study

**Figure 3:**
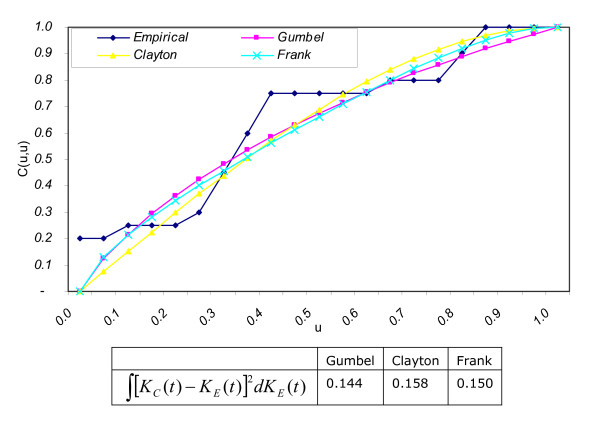
Which copula fits the best?.

Three copulas of the Archimedean family namely Gumbel, Clayton, Frank and an empirical copula [16-19] are estimated from the aortic regurgitation patients' data. These copulas are shown in Figure 3. Values of the non-parametric distance measure *DM*: ∫ [*K*_*c*_(*t*) - *K*_*E*_(*t*)]^2^*dK*_*E*_(*t*) for the Gumbel, Clayton and Frank copulas are 0.1440, 0.1580 and 0.1500 respectively. Thus, Gumbel copula is the best fit to model the given data. Monte Carlo simulations are performed to replicate datasets 50, 100, 150, 200, 250 and 300 times by implementing the algorithm to simulate bivariate data from the Gumbel copula.

The estimated prediction model and 95% confidence intervals are given in Table 4. The prediction regression model for the post-operative ejection fraction using Gumbel copula and based on 300 simulations is:

**Table 4:** Estimated prediction models and 95% confidence intervals.

	Intercept	Slope (b)	Standard Error (b)	95% Lower Confidence Interval	95% Upper Confidence Interval	Confidence Interval Width
Correlation model	-0.0658	0.8403	0.2097	0.3998	1.2808	0.8810
Gumbel model simulations						
50	-0.0768	0.8560	0.1913	0.4541	1.2579	0.8038
100	-0.0996	0.8974	0.1918	0.4945	1.3002	0.8057
150	-0.0938	0.8886	0.1914	0.4866	1.2907	0.8041
200	-0.0908	0.8855	0.1925	0.4812	1.2898	0.8087
250	-0.0965	0.8963	0.1931	0.4906	1.3020	0.8114
300	-0.0933	0.8907	0.1950	0.4810	1.3003	0.8193

Post-operative ejection fraction^copula ^= -0.0933 + 0.8907 × (Pre-operative ejection fraction); p = 0.00008; 95% confidence interval: (0.4810, 1.3003).

Since patient's age may be an important risk factor, we have included age as another predictor in the model. The estimated parameters of the age-adjusted copula based model and correlation based models are summarized in Table 5. Both prediction models indicate highly significant predictive power (R-values are 0.7010 and 0.7650 for correlation and copula based models respectively). The regression coefficient of age in both models is not significant (p-values being 0.2120 for correlation model and 0.2610 for copula model).

**Table 5:** Estimated prediction model adjusted for age and 95% confidence intervals.

	Correlation model	Gumbel model
Intercept	-0.1210	-0.1300
Slope coefficient of age (B_A_) (p-value)	0.0010 (0.4230)	0.0010 (0.5220)
Slope coefficient of pre-operative EF (B_E_) (p-value)	0.8400 (0.0010)	0.8550 (0.0010)
95% Lower confidence Interval for B_A_	-0.0020	-0.0020
95% Upper confidence for B_A_	0.0040	0.0030
95% Lower confidence for B_E_	0.3940	0.4160
95% Upper confidence for B_E_	1.2870	1.2940
R	0.7010	0.7650

**Figure 4:**
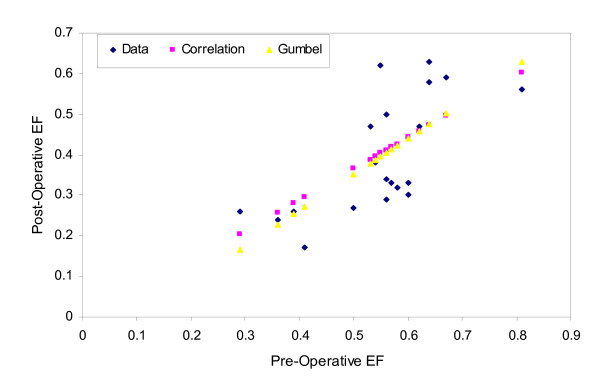
Predicted post-operative ejection fractions based on the copula- and correlation- based prediction models.

For comparison, predicted values of the post-operative ejection fractions from both copula and correlation based prediction models and actual data are shown in Figure 4. The percent absolute prediction errors for the lower pre-operative ejection fractions from the copula and correlation methods are given in Table 6. It is clear from Table 6 that prediction errors due to the copula method are smaller than those based on the correlation method. It is therefore demonstrated that the copula is a more appropriate dependence measure capable of modeling asymmetrical tails whereas correlation is not appropriate to model skewed data.

**Table 6:** Percent absolute prediction errors in the lower tail from copula and correlation models.

Pre-operative Ejection Fraction	% Absolute prediction errors (correlation model)	% Absolute prediction errors (copula model)
0.36	1.83	1.26
0.39	2.13	0.59
0.41	12.66	10.19
0.50	9.54	8.21
0.54	1.60	0.77

Further, it may be noted that estimates, standard errors and width of confidence intervals from 50,100,150,200,250 and 300 copula simulations in Table 4 are very close. Thus, the proposed copula based prediction method does not require a large number of simulations to attain consistent estimates.

### Validation using bootstrap independent data set

To validate the prediction model we were unable to obtain an independent dataset from the same population. Alternatively we have simulated fifty independent datasets by sampling with replacement from our dataset (bootstrap method). Such an approach is recommended for simulating independent datasets for methodological validation while analyzing small datasets. We found precision coefficient ryy^
 MathType@MTEF@5@5@+=feaafiart1ev1aaatCvAUfKttLearuWrP9MDH5MBPbIqV92AaeXatLxBI9gBaebbnrfifHhDYfgasaacH8akY=wiFfYdH8Gipec8Eeeu0xXdbba9frFj0=OqFfea0dXdd9vqai=hGuQ8kuc9pgc9s8qqaq=dirpe0xb9q8qiLsFr0=vr0=vr0dc8meaabaqaciaacaGaaeqabaqabeGadaaakeaacqWGYbGCdaWgaaWcbaGaemyEaKNafmyEaKNbaKaaaeqaaaaa@314B@ to be 0.8363 (p < 0.0001) indicating that the observed and predicted measurements have a strong association. The estimate of concordance statistic ρyy^
 MathType@MTEF@5@5@+=feaafiart1ev1aaatCvAUfKttLearuWrP9MDH5MBPbIqV92AaeXatLxBI9gBaebbnrfifHhDYfgasaacH8akY=wiFfYdH8Gipec8Eeeu0xXdbba9frFj0=OqFfea0dXdd9vqai=hGuQ8kuc9pgc9s8qqaq=dirpe0xb9q8qiLsFr0=vr0=vr0dc8meaabaqaciaacaGaaeqabaqabeGadaaakeaaiiGacqWFbpGCdaWgaaWcbaGaemyEaKNafmyEaKNbaKaaaeqaaaaa@31A5@ is 0.7722 (p = 0.0224) for the copula model and 0.7237 (p = 0.0604) for the correlation model. The predictions and observed measurements are therefore concordant for both models. The estimates of accuracy coefficient γyy^
 MathType@MTEF@5@5@+=feaafiart1ev1aaatCvAUfKttLearuWrP9MDH5MBPbIqV92AaeXatLxBI9gBaebbnrfifHhDYfgasaacH8akY=wiFfYdH8Gipec8Eeeu0xXdbba9frFj0=OqFfea0dXdd9vqai=hGuQ8kuc9pgc9s8qqaq=dirpe0xb9q8qiLsFr0=vr0=vr0dc8meaabaqaciaacaGaaeqabaqabeGadaaakeaaiiGacqWFZoWzdaWgaaWcbaGaemyEaKNafmyEaKNbaKaaaeqaaaaa@318C@ are 0.9233 and 0.8654 for copula and correlation models respectively.

## Discussion

It is documented [2-4] that in prediction models the Pearson's linear correlation coefficient is not a complete and accurate description of dependence structure between dependent and predictor variables even when there exists a straight-line relationship between them. An alternative method is to model the dependence structure using copulas which overcomes the limitations of correlation. Copulas are functions that join multivariate distribution functions to their one-dimensional marginal distribution functions. Copulas allow modeling of both linear and non-linear dependence. Through copulas any choice of marginal distribution functions can be used and extreme endpoint distributions can be modeled.

The copula-based approach to prediction modeling in clinical research methodology is described and is illustrated by estimating the prediction model for post-operative ejection fraction given the pre-operative ejection measurements from an aortic regurgitation patients study. The approach provides flexibility in modeling and simulating datasets because many families of copulas are known to exist in the literature. It may be noted that copula based methodology is general, since it is applicable to model data with discrete, continuous and dichotomous outcomes. However a note of caution is about the evaluation of the method based on a small data set. A more rigorous validation should be based on an independent sample taken from the population.

There appears to be connections of copulas to other nonparametric association statistics like c-statistic which are defined in terms of concordant (C) and discordant (D) pairs. One such relationship between the Gumbel copula parameter and concordant-discordant pairs is shown to exist.

## Conclusion

We emphasize that the commonly used Pearson's linear correlation coefficient is not a complete description of dependence structure even when there is a straight-line relationship between two random variables. An alternative copula-based methodology for prediction models in clinical research is described. The proposed copula-based model is capable of modeling the behavior of skewed data whereas correlation model is not appropriate for asymmetrical tails. The main statistical advantage of copulas is in replicating datasets through simulation with any type of marginal distributions.

## Competing interests

The author(s) declare that they have no competing interests.

## Authors' contributions

PK conceived of the study problem. MMS participated in formalizing study and providing data. Both authors carried out calculations. Both authors read and approved the final manuscript.

## Pre-publication history

The pre-publication history for this paper can be accessed here:



## References

[B1] Freedman AN, Seminara D, Gail MH, Hartge P, Colditz GA, Ballard-Barbash R, Pfeiffer RM (2005). Cancer Risk Prediction Models: A Workshop on Development, Evaluation, and Application. Journal of the National Cancer Institute.

[B2] Embrechts P, Mcneil AJ, Straumann D, Dempster M, Moffatt HK (1999). Correlation and dependence in risk management: properties and pitfalls. Risk Management: Value at Risk and Beyond.

[B3] Frees EW, Valdez E (1998). Understanding relationships using copulas. North American Actuarial Journal.

[B4] Schweizer B, Wolff EF (1981). On nonparametric measures of dependence for random variables. Annals of Statistics.

[B5] Sklar A (1959). Functions de repartition a n dimensions et leurs merges. Publ Inst Statist Univ Paris.

[B6] Nelson RB (1999). An Introduction to Copulas.

[B7] Genest C, Rivest L (1993). Statistical inference procedures for bivariate Archimedean copulas. Journal of the American Statistical Association.

[B8] Joe H (2005). Parametric families of multivariate distributions with given marginals. Journal of Multivariate Analysis.

[B9] Gross AJ, Lam CF (1981). Paired observations from a survival distribution. Biometrics.

[B10] Marshall AW, Olkin I (1988). Families of multivariate distributions. Journal of the American Statistical Association.

[B11] Fisher LD, van Belle G (1993). Biostatistics- A Methodology for the Health Sciences.

[B12] Schweizer B, Dall'Aglio G, Kotz S, Salinetti G (1991). Thirty years of copulas. Advances in Probability Distributions with Given Marginals.

[B13] Melchiori MR (2003). Which Archimedean copula is the right one?. Yield Curve.

[B14] Financial Risk Management. http://www.riskglossary.com/papers/Copula.zip.

[B15] Lin LI (1989). A concordance correlation coefficient to evaluate reproducibility. Biometrics.

[B16] Gumbel EJ (1960). Bivariate exponential distributions. Journal of the American Statistical Association.

[B17] Gumbel EJ (1960). Distributions des valeurs extremes en plusiers dimensions. Publ Inst Statist Univ Paris.

[B18] Clayton DG (1978). A model for association in bivariate life tables and its applications in epidemiological studies of familial tendency in chronic disease incidence. Biometrika.

[B19] Frank MJ (1979). On the simultaneous associativity of F(x, y) and x + y -F(x, y). Aequationes Math.

